# Has the Health Insurance Coverage of Scaling Contributed Positively to Periodontal Health in Korea?

**DOI:** 10.3390/ijerph17228537

**Published:** 2020-11-17

**Authors:** Jin-Sun Choi

**Affiliations:** Department of Preventive and Public Health Dentistry, College of Dentistry, Gangneung-Wonju National University & Research Institute of Oral Science, Gangneung-si 25457, Korea; jjcjsa@gmail.com; Tel.: +82-33-640-2794

**Keywords:** insurance coverage, periodontal health, scaling

## Abstract

This study aimed to evaluate the effectiveness of the health insurance coverage of dental scaling (introduced in 2013) using the Community Periodontal Index of Treatment Needs parameter among Korean adults aged 20 years or older. We used the Korea National Health and Nutrition Examination Survey data from before and after 2013 to analyze the statistical significance and associations of the covariates with the prevalence of healthy periodontal tissues, prevalence of people in need of scaling, and prevalence of periodontal diseases. The results showed that the prevalence of healthy periodontal tissues increased by 4.9% (from 34.2% to 39.1%), the number of people in need of scaling decreased by 5% (from 65.9% to 60.9%), and the prevalence of periodontal diseases increased by 7.2% (from 23.4% to 30.6%). Moreover, after the scaling coverage policy, the odds ratio of the prevalence of healthy periodontal tissues was 1.10 times higher, the prevalence of the need for scaling was 1.5 times higher, and the prevalence of periodontal diseases was 0.90 times lower. Therefore, the state should formulate policies that provide dental biofilm management through a disclosing agent, impart education about oral hygiene, and develop a health management system that enables the concurrent management of periodontal diseases and systemic diseases.

## 1. Introduction

Oral diseases are among the most common non-communicable diseases (NCDs) worldwide. As oral diseases are related to the same risk factors associated with many NCDs, their management and treatment are paramount [1]. Among the various oral diseases, periodontal diseases are particularly common, ranking 11th among the 30 most burdening diseases on a global scale [2]. In Korea, gingivitis and periodontal diseases were the second most common diseases from 2011 to 2018 and became the most common diseases in 2019 [3].

To reduce the growing number of patients suffering from periodontal diseases, the Korean government has launched health insurance coverage for dental scaling, a procedure that removes the causes that lead to the progression of gingivitis to a periodontal disease.

In Korea, the national health insurance system is a social insurance system in which people pay their monthly insurance premiums. The National Health Insurance Services (NHIS) manages and operates the system and provides insurance coverage, thereby reducing the burden of treatment costs for patients and helping them to seek healthcare services when they need them [4]. In other words, for insurance-covered treatments, people are only subject to out-of-pocket costs, with the remaining costs paid by the government. While the scope of health insurance coverage for dental care varies by age group, root canal treatment, extraction of a tooth, dental sealants, scaling, and dentures and implants for the elderly are included in the health insurance coverage [5].

The Korean scaling insurance was launched in 2013 for adults aged 20 years or older, covering one scaling per year. Hence, the individuals’ out-of-pocket cost, which used to be the total sum of treatment costs, was reduced by about three times. In 2017, the eligible age for insurance for adults was reduced to 19 years [5]. However, since the launch of the scaling coverage, the rate of dental scaling has increased steadily [6,7]. Moreover, it has been reported that the corresponding policy has increased the use of outpatient scaling care [8].

Given this context, the need to assess the impact of the increasing dental scaling rate on the actual status of periodontal health in Korea is currently under discussion. Previous studies have shed light on the need to investigate the therapeutic effects of dental scaling and the treatment of periodontal disease to determine the effectiveness of dental scaling as a prophylactic treatment [6]. These studies have reported that the prevalence of periodontal diseases must be investigated to assess the contribution of the health insurance system [9,10]. It has also been reported that more long-term assessments are needed to examine the contribution of periodic dental scaling to reducing the prevalence of periodontal diseases [11].

Previous studies have reported changes in students’ dental sealant utilization by health insurance in Korea and whether NHI’s dental sealant contributes to dental caries [12]. To sum up this situation, it is thought that research on the increasing use of dental scaling after health insurance as well as research to confirm the change in dental health conditions of the people after the policy [13,14] will be needed.

Meanwhile, the scaling offered under Korea’s health insurance system is referred to as “pre-stage treatment for removing causes and surgical treatment of periodontal diseases for treating gingivitis and periodontal diseases” [5]. No set standards have been defined for scaling. Thus, dentists and dental hygienists usually choose to scale with ultrasound scalers, except in situations in which their use might be restricted. In situations involving the use of a greater number of ultrasonic scalers, it is essential to check how the scaling method available under Korea’s scaling policy contributes to the dental health of the population. While some prior studies have called it effective in controlling infection in periodontitis patients regardless of the type of scaler [15], another study found that when the initial probing pocket depth was ≥6 mm, manual subgingival scaling was superior to ultrasound scaling, resulting in a decrease in the probing pocket depth and an increase in the clinical attachment loss [16].

The Korea National Health and Nutrition Examination Survey (KNHANES) evaluates the condition of periodontal tissue using a parameter known as Community Periodontal Index of Treatment Needs (CPITN), with reference to the World Health Organization (WHO) guidelines [17] for oral health survey. The WHO also compares periodontal conditions across countries using CPITN [18]. Previous studies have reported that CPITN provides a practical means to assess the need for periodontal treatment [19,20].

Thus, the data from the National Health and Nutrition Survey are appropriate to identify periodontal conditions. Therefore, this study uses the National Health and Nutrition Survey data and CPITN indicators to identify how much the Korean people’s dental scaling insurance policy contributes to periodontal conditions and use it as basic data to monitor periodontal conditions according to the scaling coverage.

## 2. Methods

### 2.1. Data and Participants

This study used raw data from KNHANES, which were obtained from the program’s website after obtaining permission. The purpose of KNHANES is to generate the health indices needed to evaluate whether Korea’s health policies and projects are effectively delivered by reviewing people’s health and nutritional status and trends [21]. For KNHANES, a complex sampling design named multi-level stratified clustered probability sampling is used to increase the sample’s representativeness and the accuracy of the estimation [22]. The KNHANES oral health examination was performed at a mobile examination center by a dentist who had completed practical training for quality control [23].

In field training for quality control of oral examinations, Kappa values were calculated to verify the consistency of oral test results for the reliability of the investigators [24].

The data used in this study were collected through an oral examination. Raw data were selected with reference to July 2013, the date on which the health insurance coverage for dental scaling was launched. For the pre-coverage period, we selected data from 2010 and 2012, which were the most recent data available before 2013. Unfortunately, data from 2011 were not included, as they did not contain information about the population’s periodontal tissue status [25]. Post-coverage data should be from the year after the implementation of the policy, as opposed to immediately after implementation, when the implemented policy has become stabilized. Thus, data from 2016 to 2018 (three years after the implementation of the policy) were selected as the post-coverage data. Moreover, the age limit for the analysis was set at 20 years or older, which is the eligible age for the scaling coverage. Only the participants who responded to all variables utilized in the analysis were included. As a result, 10,802 and 12,245 people before and after scaling coverage, respectively, were included in the final analysis.

The KNHANES was conducted upon approval from the Korea Centers for Disease Control and Prevention Institutional Review Board (KCDC IRB). (NO: 2010-02CON-21-C, 2012-01EXP-01-2C, 2018-01-03-P-A). The approval numbers for 2016 and 2017 are not available because the survey was considered research conducted by the government for public welfare and was, therefore, exempted from the IRB review requirement [25].

### 2.2. Procedures

The CPITN for this study was generated using the “periodontal tissue status” code under the “oral examination” category of KNHANES [26]. For the periodontal examination, the mouth was divided into sextants: upper right posterior (#18–14), upper right anterior (#13–23), upper left posterior (#24–28), lower right posterior (#36–38), lower anterior (#33–43), and lower left posterior (#44–48). Here, only the upper teeth 16–17, 11, and 26–27, and the lower teeth 36–37, 31, and 46–47 were examined to generate the scores for the periodontal tissue examination. CPI probe was used for periodontal condition inspection apparatus. The scoring criteria were as follows: 0 for healthy periodontal tissue, 1 for bleeding periodontal tissue, 2 for periodontal tissue with calculus, 3 for periodontal tissue with pocketing of 4–5 mm, and 4 for periodontal tissue with pocketing ≥ 6 mm [27]. The dependent variables, namely the prevalence of healthy periodontal tissue, prevalence of the need for scaling, and prevalence of periodontal disease, were generated using the periodontal examination scores generated. Each variable was defined as follows:

1) Healthy periodontal tissue: a score of 0 for all sextants [28];
2) Need for scaling: at least one sextant with a score of 2, 3, or 4 [28];
3) Presence of periodontal disease: at least one sextant with a score of 3 or 4 [25].

Covariates were gender (male, female), age (20–64 years, ≥65 years), region (urban, rural), and household income (1:lowest < 2 < 3 < 4:highest) in demographics; oral examination in the past year (yes, no) and tooth brushing frequency (<2, ≥2) in oral health behaviors; and smoking status (past smoker, current non-smoker/current smoker) in health behaviors.

All results were computed by considering a weighted complex sample model. The statistical significance of the covariates with the prevalence of healthy periodontal tissues, prevalence of the need for scaling, and prevalence of periodontal disease among adults aged 20 years or older were analyzed using a complex sample chi-square test. Moreover, the association between time (health insurance coverage) and dependent variables after adjusting for covariates was analyzed using complex sample logistic regression. Statistical analyses were performed using the Stata package (Stata Statistical Software: Release 13. College Station, StataCorp LP, TX, USA).

## 3. Results

Table 1 shows the numbers and weighted percentages of gender, age, region, household income, oral examination in the past year, tooth brushing frequency, and current smoking status. The numbers of adults aged 20 years or older included in the final analysis were 10,802 (100.0%) before scaling coverage and 12,245 (100.0%) after scaling coverage.

Table 2 shows the changes in the prevalence of healthy periodontal tissues according to the implementation of the Korean health insurance coverage for dental scaling for adults. The rate of healthy periodontal tissues was 34.2% before coverage and 39.1% after coverage, showing an increase of 4.9% (*p* < 0.001). The percentage of healthy periodontal tissues was higher among women than among men and higher among those aged 20–64 years than among those aged ≥65 years, both before and after scaling coverage. This percentage was also higher in the urban region than in the rural region and higher with increasing household income. The percentage of healthy periodontal tissues was also higher among those who had oral examinations in the past year, among those who brush their teeth more frequently (≥ two times per day), and among past smokers or nonsmokers than among current smokers. The differences between all variables were statistically significant (*p* < 0.001).

Table 3 shows the changes in the prevalence of need for scaling according to health insurance coverage for dental scaling in the Korean adult population. The prevalence of the need for scaling decreased by 5.0%, from 65.9% before scaling coverage to 60.9% after scaling coverage (*p* < 0.001). The percentage was higher among men than women and higher among those aged ≥65 years than among those aged 20–64 years, both before and after scaling coverage. The percentage was also higher in the rural region than in the urban region and was higher with decreasing household income. It was also higher among those who had no oral examination in the past year, those who brush their teeth less frequently (< two times per day), and among current smokers rather than past smokers or nonsmokers. The differences between all variables were statistically significant (*p* < 0.001).

Table 4 shows the changes in the prevalence of periodontal disease according to health insurance coverage for dental scaling in the Korean adult population. The prevalence of periodontal diseases increased by 7.2%, from 23.4% before scaling coverage to 30.6% after scaling coverage (*p* < 0.001). The prevalence was higher among men than women and higher among those aged ≥65 years than among those aged 20–64 years, both before and after scaling coverage. The prevalence was also higher in the rural region than in the urban region and higher with decreasing household income. It was also higher among those who had no oral examination in the past year, among those who brush their teeth less frequently (< two times per day), and among current smokers rather than past smokers or nonsmokers. The differences between all variables were statistically significant (*p* < 0.001).

Table 5 shows the association between times before and after scaling coverage and periodontal health. The launch of the scaling coverage was strongly associated with an increase in the prevalence of healthy periodontal tissues (OR = 1.10), an increase in the prevalence of the need for scaling (OR = 0.90), and an increase in the prevalence of periodontal diseases (OR = 1.50) (*p* < 0.01, *p* < 0.001). After adjusting for the covariates, the statistical significance for prevalence of healthy periodontal tissues and the need for scaling decreased, while the statistical significance for prevalence of periodontal diseases was maintained.

## 4. Discussion

In this study, we confirmed that implementation of dental scaling coverage led to an increase in the percentage of adults with healthy periodontal tissues, a decrease in the percentage of adults in the need for scaling, and an increase in the percentage of adults with periodontal disease.

In this study, we examined the effectiveness of the health insurance coverage of dental scaling launched in 2013 by observing changes in the CPITN before and after the implementation of scaling coverage.

The percentage of Korean adults with healthy periodontal tissues increased by 4.9% with the launch of scaling coverage, from 34.2% before the policy to 39.1% after the policy (*p* < 0.001). The percentage of Korean adults in need of scaling decreased by 5%, from 65.9% before the policy to 60.9% after the policy (*p* < 0.001). Moreover, the implementation of scaling coverage was associated with an increased (OR = 1.10) percentage of healthy periodontal tissues and decreased (OR = 0.90) need for scaling (*p* < 0.01, *p* < 0.001). The percentage of people in need of scaling was 60.9%, showing that more than half of the population needs scaling. Considering that the targeted scaling rate among adults (35–44 years) in Korea’s Health Plan is 50% in 2020 [29], consistent effort is needed to lower the need for scaling in the coming years. Moreover, these results suggest that the implemented policy is likely to produce targeted outcomes. Preceding studies also point out that insurance coverage for dental scaling has a positive effect on middle-aged and elderly people’s access to dental disease prevention treatment [30]. Therefore, the state should continue to have an interest in the management of periodontal diseases and consider public health approaches to pursue improved methods of preventing and managing periodontal diseases [31].

The prevalence of periodontal diseases rose by 7.2%, from 23.4% before the policy to 30.6% after the policy. Thus, the implementation of scaling coverage was found to be strongly associated with an increased (OR = 1.50) prevalence of periodontal diseases (*p* < 0.001). The result that the number of people with periodontal disease is on the rise suggests that the scaling coverage policy has limitations in preventing periodontal diseases. One of the causes of periodontal diseases is dental biofilms, and management of biofilms [32], oral health education for patients, and continuous maintenance and care are crucial for preventing periodontal diseases [33]. However, current dental scaling procedures are generally focused on removing dental calculus, and they rarely include removal of biofilms and tooth brushing education for maintenance and management following calculus removal, which are not covered by health insurance benefits. Therefore, to prevent periodontal diseases by removing calculus, customized preventive treatment strategies should be provided by expanding coverage to care activities such as dental plaque management using a disclosing solution (which visualizes the biofilm and thus helps remove it), customized toothbrushing education, and professional toothbrushing training. Mensi et al. reported that using a plaque disclosing agent would lead to better removal of biofilms [34], and Tonetti et al. reported that repetitive and individualized oral hygiene instruction and professional mechanical plaque removal are important components of a preventive program [35]. Moreover, Matthews et al. recommended that dental clinicians should check whether patients can remove the biofilm when providing prevention and treatment of periodontal diseases [36].

Furthermore, the association between periodontal and systemic diseases must also be discussed. The prevalence of chronic diseases (e.g., hypertension, diabetes mellitus, hypertriglyceridemia, and hypercholesteremia) is continuously rising in Korea [37]. The association between periodontal and systemic diseases has been observed in many previous studies [38,39,40,41], and hence, the importance of comprehensively managing systemic and periodontal diseases is growing. In particular, factors such as diabetes increase the risk for periodontal diseases and may exacerbate their severity [42,43]. In other words, a health-promoting system that concurrently manages the common risk factors of systemic diseases and periodontal diseases needs to be established. The World Dental Federation (FDI) has argued that oral diseases should be managed together with chronic diseases and has proposed oral health-promoting strategies [44]. The WHO designed an approach to integrate the prevention of non-communicable chronic diseases, prevention of oral diseases, and enforcing global strategies in all regions around the world [18]. Prasad et al. argued that oral health management must be developed as an essential component of primary health care [45]. Petersen et al. also suggested that national public health policies to control and prevent periodontal diseases should encompass both oral health-promoting approaches that address general risk factors as well as integrated disease prevention strategies [46]. Furthermore, Son et al. stressed that to improve and maintain dental and periodontal health, patients with chronic diseases (characterized by risk factors common with those of periodontal diseases) such as metabolic syndrome, diabetes mellitus, cardiovascular disease, and obesity must be distinguished and the relationship between periodontal diseases and systemic illnesses must be considered [47]. Due to its transition as an ageing society and the dramatic spike in chronic diseases, the Korean government preliminarily implemented the “Primary care chronic disease management project” in 2019, but oral diseases are not included in the scope of chronic diseases. To keep abreast of the global chronic disease management paradigm, Korea should also modify and update its current health management system to enable concurrent management of systemic diseases, lifestyle, and oral health.

This study is significant in that it assessed periodontal diseases using a clinical index following the implementation of health insurance coverage of dental scaling. However, simple observational studies make it difficult to identify the causal relationship associated with periodontal health conditions and have limitations in identifying practical policy effects. Therefore, studies are needed to identify causality considering covariates that may affect the increasing trend of periodontal disease and to use time-series analysis or a change-in-diffusion approach. Moreover, comparisons between indicators that can measure periodontal conditions should enable results to be produced with more reasonable indicators.

## 5. Conclusions

We confirmed that after the dental removal health insurance policy, the number of people with healthy teeth and periodontal disease increased, and the number of people with dental floss decreased. To have a more positive effect on reducing periodontal disease sufferers, in addition to the health insurance coverage of dental scaling, policies that can provide dental plaque management using a discoloring agent and oral hygiene education to strengthen individuals’ oral hygiene would be needed. Furthermore, a health management system that enables concurrent management of systemic and periodontal diseases must be established. Moreover, it is necessary to identify the factors that affect the policy through in-depth analysis and to monitor the effectiveness of the policy over the long term.

## Figures and Tables

**Table 1:** Characteristics of subjects.

Classification		Before		After	
		N	Weighted (%)	N	Weighted (%)
Total		10,802	100.0	12,245	100.0
Gender	Male	4569	49.7	5315	41.8
	Female	6233	50.3	6930	58.2
Age	20–64 years	8485	87.7	9410	77.4
	≥65 years	2317	12.3	2835	22.6
Region	Urban	8647	80.4	10,125	84.5
	Rural	2155	19.6	2120	15.5
Household income	1 (lowest)	1937	15.1	2203	17.5
	2	2771	26.6	2986	24.1
	3	3018	29.6	3449	28.2
	4 (highest)	3076	28.7	3607	30.2
Oral examination in the past year	No	8221	76.4	7770	63.1
	Yes	2581	23.6	4475	36.9
Tooth brushing frequency	<2	1241	11.1	1072	8.2
	≥2	9561	88.9	11,173	91.8
Smoking status	Past smoker/ Current non-smoker	8631	73.9	10,054	82.7
	Current smoker	2171	26.1	2191	17.3

**Table 2:** Changes in the prevalence of healthy periodontal tissues according to health insurance coverage for dental scaling.

Classification		Before				After			
		Total	N	Weighted (%)	*p* Value	Total	N	Weighted (%)	*p* Value
Total		10,802	3584	34.2		12,245	4634	39.1	
Gender	Male	4569	1230	29.0	<0.001	5315	1702	33.3	<0.001
	Female	6233	2354	39.3		6930	2932	43.3	
Age	20–64 years	8485	2977	35.3	<0.001	9410	3704	40.7	<0.001
	≥65 years	2317	607	25.7		2835	930	33.8	
Region	Urban	8647	3088	36.6	<0.001	10,125	4076	41.4	<0.001
	Rural	2155	496	24.3		2120	558	26.8	
Household income	1 (lowest)	1937	503	27.7	<0.001	2203	696	32.9	<0.001
	2	2771	859	32.6		2986	1065	36.5	
	3	3018	1081	36.0		3449	1366	41.2	
	4 (highest)	3076	1141	37.1		3607	1507	43.0	
Oral examination in the past year	No	8221	2502	31.2	<0.001	7770	2625	34.5	<0.001
	Yes	2581	1082	43.6		4475	2009	47.1	
Tooth brushing frequency	<2	1241	270	21.8	<0.001	1072	301	28.5	<0.001
	≥2	9561	3314	35.7		11,173	4333	40.1	
Smoking status	Past smoker/ Current non-smoker	8631	3054	37.0	<0.001	10,054	4050	41.6	<0.001
	Current smoker	2171	530	26.1		2191	584	27.3	

The data were analyzed by Complex samples chi-square test.

**Table 3:** Changes in the prevalence of need for scaling according to health insurance coverage for dental scaling.

Classification		Before				After			
		Total	N	Weighted (%)	*p* Value	Total	N	Weighted (%)	*p* Value
Total		10,802	7218	65.9		12,245	7611	60.9	
Gender	Male	4569	3339	71.0	<0.001	5315	3613	66.7	<0.001
	Female	6233	3879	60.7		6930	3998	56.7	
Age	20–64 years	8485	5508	64.7	<0.001	9410	5706	59.3	<0.001
	≥65 years	2317	1710	74.3		2835	1905	66.2	
Region	Urban	8647	5559	63.4	<0.001	10,125	6049	58.6	<0.001
	Rural	2155	1659	75.7		2120	1562	73.2	
Household income	1 (lowest)	1937	1434	72.3	<0.001	2203	1507	67.2	<0.001
	2	2771	1912	67.4		2986	1921	63.5	
	3	3018	1937	64.0		3449	2083	58.9	
	4 (highest)	3076	1935	62.9		3607	2100	57.0	
Oral examination in the past year	No	8221	5719	68.8	<0.001	7770	5145	65.6	<0.001
	Yes	2581	1499	56.4		4475	2466	52.9	
Tooth brushing frequency	<2	1241	971	78.2	<0.001	1072	771	71.5	<0.001
	≥2	9561	6247	64.3		11,173	6840	59.9	
Smoking status	Past smoker/ Current non-smoker	8631	5577	63.0	<0.001	10,054	6004	58.4	<0.001
	Current smoker	2171	1641	73.9		2191	1607	72.7	

The data were analyzed by Complex samples chi-square test.

**Table 4:** Changes in the prevalence of periodontal diseases according to health insurance coverage for dental scaling.

Classification		Before				After			
		Total	N	Weighted (%)	*p* Value	Total	N	Weighted (%)	*p* Value
Total		10,802	2876	23.4		12,245	3844	30.6	
Gender	Male	4569	1524	28.2	<0.001	5315	2043	37.8	<0.001
	Female	6233	1352	18.7		6930	1801	25.5	
Age	20–64 years	8485	1914	20.9	<0.001	9410	2485	25.6	<0.001
	≥65 years	2317	962	41.5		2835	1359	47.7	
Region	Urban	8647	2059	20.7	<0.001	10,125	2947	28.4	<0.001
	Rural	2155	817	34.7		2120	897	42.9	
Household income	1 (lowest)	1937	757	33.6	<0.001	2203	958	42.8	<0.001
	2	2771	772	24.8		2986	1044	34.5	
	3	3018	707	21.2		3449	973	27.7	
	4 (highest)	3076	640	19.0		3607	869	23.2	
Oral examination in the past year	No	8221	2279	24.2	<0.001	7770	2634	33.3	<0.001
	Yes	2581	597	20.8		4475	1210	26.0	
Tooth brushing frequency	<2	1241	510	36.6	<0.001	1072	470	44.3	<0.001
	≥2	9561	2366	21.8		11,173	3374	29.4	
Smoking status	Past smoker/ Current non-smoker	8631	2148	21.7	<0.001	10,054	2906	28.3	<0.001
	Current smoker	2171	728	28.2		2191	938	41.8	

The data were analyzed by Complex samples chi-square test.

**Table 5:** Association between insurance coverage implementation and periodontal health.

Classification	The Prevalence of Healthy Periodontal Tissues		The Prevalence of Need for Scaling		The Prevalence of Periodontal Diseases	
	Crude	Adjusted	Crude	Adjusted	Crude	Adjusted
	OR 95% (CI)	OR 95% (CI)	OR 95% (CI)	OR 95% (CI)	OR 95% (CI)	OR 95% (CI)
Before	1.00	1.00	1.00	1.00	1.00	1.00
After	1.23 (1.16–1.32) ***	1.10 (1.03–1.17) **	0.80 (0.75–0.86) ***	0.90 (0.84–0.97) **	1.44 (1.34–1.54) ***	1.50 (1.40–1.62) ***

** *p* < 0.01, *** *p* < 0.001. The data were analyzed by a logistic regression. Adjusted for gender, age, region, household income, oral examination in the past year, tooth brushing frequency, smoking status.
